# Factors affecting implementation of interventions for oral health, substance use, smoking and diet for people with severe and multiple disadvantage: a community-based qualitative study in England

**DOI:** 10.1136/bmjph-2023-000626

**Published:** 2024-05-02

**Authors:** Neha Jain, Emma A Adams, Emma C Joyes, Gillian McLellan, Martin Burrows, Martha Paisi, Laura J McGowan, Lorenzo Iafrate, David Landes, Richard G Watt, Falko F Sniehotta, Eileen Kaner, Sheena E Ramsay

**Affiliations:** 1 Newcastle University Population Health Sciences Institute, Newcastle Upon Tyne, UK; 2 Inclusive Insights, Bournemouth, UK; 3 University of Plymouth School of Nursing and Midwifery, Plymouth, UK; 4 NHS England and Improvement, Newcastle Upon Tyne, UK; 5 Department of Epidemiology and Public Health, University College London, London, UK

**Keywords:** Public Health, Translational Science, Biomedical, Community Health

## Abstract

**Background:**

Populations facing severe and multiple disadvantage (SMD), co-occurring homelessness, substance use and repeat offending have high levels of physical and mental ill-health. Poor oral health is one of the most common health problems in this population and is closely linked with substance use, smoking and poor diet. Issues related to the implementation of interventions among SMD populations to address oral health and related health behaviours are poorly understood. This study aimed to understand the factors that affect implementation (relevance of setting, acceptability and adverse effects of interventions) and the sustainability of interventions targeting oral health, substance use, smoking and diet for people experiencing SMD.

**Methods:**

Between August 2021 and April 2023, interviews and focus group discussions were conducted with two groups of participants: (1) people experiencing SMD in Newcastle Upon Tyne/Gateshead and (2) frontline staff, volunteers, policymakers and commissioners from London, Plymouth and Newcastle Upon Tyne/Gateshead. Information was gathered on factors related to the implementation and acceptability of interventions related to oral health and related behaviours. The data were analysed iteratively using thematic analysis.

**Results:**

Twenty-eight people experiencing SMD (age range: 27–65 years; 21% females) and 78 service providers, commissioners and policymakers (age range: 28–72 years; 63% females) were interviewed or included in focus groups. The data were organised into three overarching themes: environmental, organisational and interpersonal factors. Environmental factors included funding and integrated services; organisational factors included inclusive services, health promotion, prevention and training healthcare providers; interpersonal factors included the presence of support workers and motivation among service providers.

**Conclusion:**

The implementation and sustainability of health interventions for people experiencing SMD are influenced by factors across environmental, organisational and interpersonal levels that interact with the inherent challenges of disadvantaged groups. The findings highlight the need for tailoring healthcare interventions according to the needs of people experiencing SMD. Further research on the implementation of diet interventions and co-producing interventions is needed.

**WHAT IS ALREADY KNOWN ON THIS TOPIC**

- People experiencing severe and multiple disadvantage (co-occurring homelessness, substance use and/or repeat offending) face worse and often interlinked negative physical and mental health. They also experience challenges when engaging with health interventions.

**WHAT THIS STUDY ADDS**

- Speaking to people experiencing severe and multiple disadvantages alongside stakeholders in policy and practice, we found that the wider social and political environment, organisational policies and relationships with service providers affect the implementation and sustainability of oral health, substance use, smoking and diet interventions.

**HOW THIS STUDY MIGHT AFFECT RESEARCH, PRACTICE OR POLICY**

- Consideration is needed for how the factors identified for improving implementation (relevance of setting, acceptability and effects of intervention) and sustainability can be adapted into current practice. Subsequently, the effectiveness of these interventions in improving health outcomes for people experiencing severe and multiple disadvantage should be investigated.

## Introduction

 Homelessness in England has risen over the last few years, with more than 3000 people sleeping rough in one night as compared with 1768 in 2010.^1^ A significant proportion of people experiencing homelessness are simultaneously affected by substance use and/or repeat offending.^2^ The co-occurrence of homelessness, substance use and repeat offending is referred to as severe and multiple disadvantage (SMD). These domains of SMD often closely relate to one another and can be causes or consequences of each other.^3 4^ SMD is commonly determined by risk factors such as socioeconomic deprivation, poverty, childhood neglect or abuse and mental illness.^5 6^ It also leads to very high levels of physical and mental ill-health, which cost the public sector in the UK around £10 billion/year.^5^

Poor oral health is one of the most common physical health issues concerning people facing SMD.^7^ The prevalence of oral health conditions such as tooth loss, tooth decay, infections and pain is very high among those experiencing SMD.^8^ Peer-led research has shown that 90% of homeless populations had a problem with their mouth, 60% experienced dental pain, and 70% have lost their teeth since becoming homeless.^9^ This invariably affects their physical and mental health, food intake, self-esteem and day-to-day functioning and can lead to stigma, social isolation and the loss of employment opportunities.^8^ Smoking, substance use and poor diet are also very prevalent in people experiencing SMD. These health behaviours are established risk factors for oral diseases and are closely interrelated with poor oral health and general health. In people experiencing SMD, poor oral health additionally leads to greater drug and alcohol use and smoking as a way of coping with dental pain and related issues of poor mental health, isolation and low self-esteem, thus creating a perpetual cycle of harm.^9^ The issues of poor oral health, substance use, smoking and diet also have common underlying risk factors, such as poverty, low education levels and unstable housing.^10^ These issues are further worsened by poor access to and engagement with services for oral health,^9 11^ substance use and mental health.^12 13^

The Local Government Association highlighted the need to improve the fragmented care currently available to SMD groups.^14^ Improving access to physical and mental health services for people experiencing SMD, including oral healthcare, is a national priority highlighted by several government and non-government agencies.^14^ However, there is limited actionable evidence for policymakers and commissioners on the implementation of interventions such that they are usable and sustainable for the people experiencing SMD. A targeted approach to interventions, in the way they are delivered to people experiencing SMD, could help ensure better uptake of services and reduce inequalities in access to interventions. Therefore, this study aimed to understand the factors that impact the implementation (relevance of setting, acceptability and adverse effects of intervention) and sustainability of interventions targeting oral health, substance use, smoking and diet for people experiencing SMD.

## Methods

We used qualitative methods to answer the aim of this study. In order to get input into our study from people with lived experience of SMD, we worked with a service user group run by a charity in Newcastle upon Tyne and Gateshead. We organised Patient and Public Involvement and Engagement (PPIE) workshops at different stages of the study, where we met with a group of 2–4 people with lived experience of SMD to get input from people with lived experience of SMD in different aspects of our study. Information sheets, choice of language used, data collection approaches and recruitment strategies were determined and shaped with their guidance. For instance, the members of the PPIE group supported designing an animated video for participant recruitment. The language and animation of this video, as well as the topic guides, were improved using feedback from the group. Following initial data collection, preliminary themes were discussed with people with lived experience before continuing with data collection.

The focus of the data collection was to explore the insights and experiences of two stakeholder groups in the implementation of interventions related to oral health, substance use, smoking and diet. The two stakeholder groups were (1) people with past or present experience of SMD and (2) support service staff and volunteers (frontline), policymakers and commissioners (service providers). People with experience of SMD included those who were aged 18 years and above and had lived experience of homelessness with other domains of SMD, including substance misuse and/or repeat offending. Service providers included those involved in the planning, commissioning or delivery of interventions related to oral health, substance use, smoking and diet for people facing SMD.

### Recruitment and data collection

Purposive sampling was used to recruit participants for both stakeholder groups, with sampling criteria being age, gender, location, role for service providers and SMD domain for people experiencing SMD. People experiencing SMD were recruited through lived experience networks, homeless drop-in centres and voluntary community sector organisations in the Newcastle upon Tyne and Gateshead areas. Service providers were recruited through existing networks, mailing lists and snowball strategies. To allow additional exploration of themes and findings and ensure the inclusion of diverse experiences, people experiencing SMD were recruited in two phases. The first data collection took place between August and October 2021 and focused on people with current or past experience with SMD. The second part of data collection for people experiencing SMD took place in April 2023 in order to further explore and validate the initial themes from the first data collection. Also, the first data collection took place during the COVID-19 pandemic, and the second data collection helped gather experiences that were not necessarily influenced by the issues of the pandemic. Service providers were recruited between October 2021 and January 2022. In order to get some wider representation from service providers on the delivery of interventions in different areas in England, we conducted data collection across Newcastle/Gateshead, London and Plymouth for this participant group.

Data were collected until saturation was reached in each individual area and participant group, and sufficient information was collected to form well-defined codes with respect to the planning, adoption and feasibility of implementing health services for oral health and associated health behaviours in people experiencing SMD. Detailed information about the sampling criteria can be found in the published protocol paper.^15^ All participants were provided with an information sheet, which was reviewed and any questions addressed prior to obtaining written or verbal consent and commencing data collection. Interviews were conducted by multiple researchers on the study team (EAA, SER, LJM, GM, MB, MP, LI and NJ) with past experience in qualitative research and data collection. Researchers met regularly during data collection to talk through any common issues encountered and to help with consistency across interviews. Interviews with service providers were conducted online using MS Teams or Zoom, whereas those with people experiencing SMD were a mix of online (Teams/Zoom), telephone and in-person discussions. Interviewers usually introduced themselves prior to commencing data collection and ensured that the participants had all their questions answered. Separate semistructured topic guides were used to interview each stakeholder group, which covered (1) what works well and less well with current services, (2) current challenges with oral health, substance use, smoking and diet interventions and (3) suggestions for how things could be improved. Topic guides for interviewing people experiencing SMD as well as the service providers are attached as onlinesupplemental appendices 1  2. People experiencing SMD received a £25 voucher as a thank you for participating. Each interview with people experiencing SMD lasted between 20 and 30 min, and those with service providers lasted 40–60 min.

This study was approved by the Faculty of Medical Sciences Research Ethics Committee, part of Newcastle University’s Research Ethics Committee (Ref: 9727/2020; 2066/9725).

### Data analysis

All the interviews were audio-recorded and transcribed verbatim. Separate interview notes were also written by each interviewer to note any specific participant characteristic or incidence during the interview that may impact the data. This study was underpinned by grounded theory.^16^ No hypothesis was set prior to collecting data, and an iterative process was followed where subsequent interviews or focus group discussions were based on emerging themes. Once data collection was completed, the data were analysed using Braun and Clarke’s approach to reflexive thematic analysis.^17^ This approach provided the flexibility to explore cultural, policy-related and programmatic challenges and solutions for more than one health condition and the health services involved. A combined inductive and deductive approach to data analysis was used; the initial phase of analysis was largely deductive, in which a codebook approach was employed to ensure that information coinciding with the aims of the project was coded, and this was followed by a more inductive analysis of the descriptive discussions with service providers and people with experience of SMD.^17^

Once the initial codes were developed, they were organised into overarching categories corresponding to the domains of the socioecological model. The socioecological model, a conceptual theory developed in the 1980s, places individual health in relation to various microlevel and macrolevel influences and how these factors interact with each other. This can include the impact of policy-level change at a macrolevel and the impact of relationships at a microlevel. The socioecological model has been adapted in various contexts of public health in the past to study health promotion, behaviour change or prevention. In our study, drawing comparison with the model and looking at the health interventions through the lens of the socioecological model allowed us to investigate the implementation of interventions comprehensively and across various levels of determinants that often interact with one another to determine the outcome.

All identifiable data were removed by a member of the research team and replaced with pseudonyms. During the initial review, the data were categorised according to a few elementary codes by one researcher who expressed the data in a manner consistent with the overall research objectives of the project. Thereafter, using the constant comparative method,^18^ all the coded data were visited and revisited to develop themes based on similarities and dissimilarities in data. The themes were then re-evaluated to create a thematic map of the analysis, and all themes were then defined with support from the lived experience group. All data were organised and reviewed using qualitative analysis software, NVivo, V.14.23.1.^19^

## Results

A total of 106 participants were interviewed through one-to-one interviews (n=89) or focus groups (n=17). Each focus group had between two and five people. Participants included 28 people with experience with SMD (current or past) and 78 frontline workers, healthcare providers and policymakers. People experiencing SMD had a range of housing situations, including living in supported accommodations or temporary accommodations (hostels), insecure housing, sleeping on a friend’s or relative’s sofa (sofa surfing) and rough sleeping.

Detailed demographic characteristics of the study participants are provided in table 1.

**Table 1:** Demographic description of study participants

Service providers (n=78)		
Age range (mean)		28–72 years (48.2 years)
Gender	Females	49 (63%)
Ethnicity	White British or other white background	62 (79.5)%
	Asian or Asian British	5 (6.4%)
	Black, African, Caribbean or Black British	3 (3.8%)
	Others	5 (6.4%)
Work sector	Local authority	11 (14%)
	Healthcare	25 (32%)
	Third sector	30 (38%)
	Policy	12 (15%)
Type of role	Frontline	30 (38.5%)
	Manager	11 (14.1%)
	Dual role (frontline and manager/manager and commissioning)	13 (16.7%)
	Commissioning/policymaker	16 (20.5%)
	Other	8 (11.5%)
**People with experience of SMD from Newcastle upon Tyne/Gateshead (n=28)**		
Age range (mean)		27–65 years (44 years)
Gender	Females	6 (21.4%)
Ethnicity	White British or other white background	27 (96.4%)
	Black, African, Caribbean or Black British	1 (3.6%)

**Figure 1:**
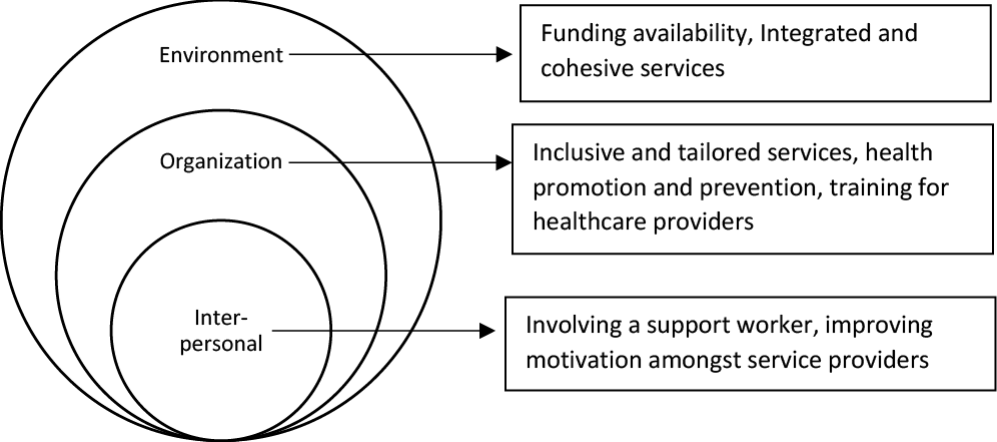
Themes and subthemes reflecting factors influencing the implementation and sustainability of health interventions for people experiencing severe and multiple disadvantage.

Identified themes were broadly grouped into the outer three domains of the socioecological model^20^: environmental factors, corresponding to wider policies, commissioning and design of health services; organisational factors, related to the implementation and delivery of health services at the organisation level and interpersonal relationships, describing the role of service providers and their interaction with people with lived experience of SMD. Subthemes are listed in figure 1 and described below.

### Environmental factors

Environmental factors included the overarching financial, social, political and cultural influences within which health interventions are delivered to people experiencing SMD. These are factors that influence the availability of resources and how different health services work and integrate to deliver health interventions to people experiencing SMD.

#### Funding availability

Study participants, mostly service providers, reported that resources, or lack thereof, were a major factor that drove the availability and implementation of health interventions for people experiencing SMD. The participants expressed that the provision of health interventions was often dependent on the way that services were being commissioned by the National Health Services (NHS) in the UK. In turn, commissioning health services was often about the health needs of the general population in a specific location, the prevalence of people experiencing SMD in that location and deciding which services were to be prioritised for the money available.

Definitely looking at the demography of the population, the influx, the flow, say onto the streets and into the settings… They’re going to be looking at obviously the need and then I’d say what are the other conflicting commissioning priorities in the area? We have to be realistic because there’s a need for many different populations and when do they feel commissioning a service would be best? (Service Provider: female, early 30s)

Lack of funding was also perceived as a major barrier for ensuring the sustainability of interventions, particularly impacting the ability to engage staff for longer durations.

Funding is the key, because if you've got a long-term funding stream, you're going to get people committed to these services. If you've got very volatile funding screens, then there’s no commitment from anybody to maintain these services… And the other issue which people get very concerned about, if they've got short-term funding is redundancy costs. So, no one’s going to be employing anybody, if they've only got funding for perhaps two-and-a-bit years, because then who is going to cover the costs of any redundancies? (Service Provider: male, late 50s)

People experiencing SMD, while having limited insights compared to service providers about funding per se, reported that there was a lack of available services for them.

There isn’t enough help out there, there isn’t enough funding and there isn’t enough support. (Person experiencing SMD: female, late 40s)

#### Integrated and cohesive services

Study participants discussed the health complexities of people experiencing SMD and the need to provide holistic care and support through links between services so that their implementation is easier in terms of cohesion between different healthcare providers. Cohesive services gave an opportunity for different case workers or healthcare providers to create a well-integrated plan and facilitate easy sharing of information for issues such as mental health and substance use, which are known to commonly co-occur.

…coming up with a joint way of working so that everybody is still able to support the person in whatever needs they have, and nothing is getting missed out. So, we’re not missing out things like their dental care or them checking in with their GP for other physical health conditions and not just focusing on maybe substance misuse or just benefits but a whole picture of what they need because people don’t just need one thing, as we all know. (Service Provider: female, age not provided)

Cohesion between various services, such as mental health and addiction or smoking cessation services and pharmacies, was reported to be fragmented, making it difficult to deliver the intervention to people experiencing SMD.

… they’d say, ‘pick your prescription up at the chemist’. Sometimes, I’d get them off the doctor. You’d go into the chemist, and they’d say, ‘we haven’t got any.’ Access to the stuff that you were being prescribed wasn’t available. You’d have to wait five days for it to come in stock. By then, you’d picked twenty fags up or a packet of tobacco, so it was a waste of time. (Person experiencing SMD: female, late 40s)

### Organisational factors

These factors describe the settings and conditions within which health interventions are delivered and can create a supportive environment for implementing health interventions. These pertain to the dynamics and policies within healthcare settings, such as hospitals, clinics or any outreach services, including their role in health promotion that can influence the sustainability of healthcare interventions for people experiencing SMD.

#### Inclusive and tailored services

Participants reported that current services sometimes did not accommodate the challenges that often came with a lack of permanent housing. For example, people experiencing SMD and without a fixed address often reported facing challenges in accessing general practitioner (GP) services because of difficulty getting registered with a GP without an address.

I needed a doctor, due to my health and obviously my mental health, my illness. I couldn’t get a doctor because I didn’t have a fixed address. (Person experiencing SMD: female, early 40s)

Study participants also reported that it was important that the services be flexible to accommodate the logistical challenges faced by people experiencing SMD. It was expressed that sometimes if the appointment for a service or a session was set up, for example, very early in the morning, it was unlikely that people experiencing SMD would be able to make it to the appointment on time. Second, consecutively missing two appointments with the GP would deregister the people from their GP practice, hence precluding them from receiving the intervention when needed. Considering the ability of the person experiencing SMD, having more flexible appointments or the availability of walk-in services was suggested to make implementing the intervention more feasible.

Pre-lockdown, it used to take a hell of a long time to get an appointment. You would only be able to do it at 8:30 AM, knowing some of our clients would never make it at 8:30 AM, and if they were five or ten minutes late, they would ban them from going in and they would have to come back another day, so they would go without a script. (Service provider: female, early 60s)

…If they offered flexibility of appointments, I may have attended, and I could have rearranged my calendar and arranged with my boss to take time off… But when I did try and get in touch with a cessation service… I couldn’t be there at the times they were open, so it didn’t work for me, and there weren’t any alternatives. (Person experiencing SMD: male, early 60s)

Long waiting times were also considered to be hindering the delivery of interventions, especially for substance use and dental problems such as infections. Delayed interventions were reported to affect abstinence from drug use and the relapse of infections.

…it does sometimes seem like it is quite a long time to be rescripted… because then they go off and use, and you wouldn’t have done that if you could have sorted it out a bit quicker. (Service provider: female, early 30s)

They did actually give me antibiotics to help bring the abscess down, and then they are telling me to register with a dentist. But I have registered with the dentist, and I still haven’t had a phone call from them, and it has been a year and a half now. (Person experiencing SMD: male, late 20s)

#### Health promotion and prevention

People experiencing SMD reported a lack of knowledge about harmful health behaviours, the presence of health interventions and general health information, which impacted their health outcomes, especially when it came to oral health. For example, people experiencing SMD reported a lack of awareness about the high sugar content of methadone (a harm-reduction drug to help manage withdrawal symptoms of heroin addiction) and suggested that prior information and knowledge about the best way to take methadone would have helped with making an informed decision.

…so, what they say is you should be making sure that you drink a glass of water beforehand, brush your teeth afterwards and then make sure you’re drinking after as well to really cleanse the palate in the mouth and gums because of the long-term effect and the short-term effect that methadone has on the teeth and gums. (Person experiencing SMD: male, early 40s)

Many service providers suggested that promoting health within and outside a clinical setting, through health education, empowerment, as well as making resources such as toothbrushes and toothpaste available, can go ‘a long way’ in creating an oral health intervention that is sustainable for people experiencing SMD. A lack of knowledge about oral health was reported to be a reason why people experiencing SMD did not seek oral health treatment or care.

Yeah, if we look at oral health promotion and preventative advice to begin with…there’s so much still to explore by in reach and outreach actually. I don’t feel we’re often doing enough; going into hostels, going into the settings, going into prisons and providing preventative advice that should be available to everybody. Also building trust through oral health promotion as well… You’re making more contacts with individuals to build trust, it just makes sense. You’re building a relationship and you’re an advocate for that clinic. (Service Provider: female, early 30s)

#### Training healthcare providers

Study participants reported that due to the complex health profiles of people experiencing SMD, particularly the presence of mental health conditions, it is difficult for healthcare providers to effectively deliver an intervention. For example, training in trauma-informed care for the entire healthcare provider team (dentists, nurses, receptionists, etc.) would be helpful in reducing the stigmatisation reported by people experiencing SMD when receiving healthcare. Study participants reported that service providers ‘did not understand’ the problems of people experiencing SMD and were constantly judged because of that. This prevented them from seeking further care.

I didn’t like some of the attitudes, especially the dental hygienists, they’re nightmares. You know, you can have a nice dentist but then they’ll send you to see the dental hygienist and they’ve got a terrible attitude because they do not understand. That’s what used to put me off as well. (Person experiencing SMD: female, late 40s)

I think also if we did have more training there would be a better understanding and less fear. I think it’s fear that drives us to not necessarily treat these patients. It’s not necessarily that we want to be unkind or not treat, it’s just that we don’t have an understanding so we judge and we presume. (Service provider: female, early 40s)

### Interpersonal factors

Interpersonal factors relate to the interaction and relationship between a service provider and people experiencing SMD, which influence the implementation of interventions and health outcomes. These factors include the role of communication, social support and trust in meeting the healthcare needs of people experiencing SMD.

#### Including a support worker

Having a support worker was reported to act as a link between health services and service users to help facilitate access to healthcare interventions. This ensured that people get the information they need and help with attending their appointments. Navigating healthcare systems was reported to be easier when a support worker was someone with lived experience of SMD.

…it was my support worker who registered me there, and she took me down. So it was quite good. It wasn’t that bad of an experience, actually. And I was glad that she was there to support me. (Person experiencing SMD: male, late 30s)

I almost needed somebody to stand by the side of me and say, ‘Today’s the day, put your patch on.’ Next day, ‘Put your patch on.’ I would never have remembered. If it was a case of if I only lived close to the doctors, which I didn’t, I could have gone there and got my patch every day. That would have been great. (Person experiencing SMD: male, late 50s)

#### Motivation among service providers

It was reported that the delivery of interventions was dependent on the way they were commissioned by the NHS in the UK. For services that were not covered under this universal health system, it was often harder to deliver them. For example, with outreach services, such as the use of mobile dental vans, it was easier to overcome issues related to access to health interventions by people experiencing SMD. However, such outreach services were not usually funded by the NHS and needed support from charitable organisations and volunteer frontline workers and be motivation driven.

I used to live next to a homeless centre … And we did discuss trying to help them actually at the clinic and there were a lot of barriers to that. And unless you really have dentists that are more or less doing it … because there’s no remuneration in the staff of the NHS system for doing this stuff. So, it has to be dentists that are saying, we’re doing this, you know, because they need it and not because we’re being remunerated for it. (Service provider: male, early 60s)

Reported common examples of services that worked well included those where there was a provision for a service provider to volunteer some hours in a week, either on weekends or otherwise, to provide health services or advice in drop-in centres or other places within the community.

He did it a couple of hours every morning and a couple of hours every night… his Saturday was spent doing it. The only day he had off was a Sunday. If they could clone him, it would be fantastic. He just went that extra mile. He really, really wanted to help. He never turned anybody down. If somebody turned up to his place, “I need an appointment.” “We haven’t got one. We’ll fit you in.” You’d always get fitted in. (Person experiencing SMD: male, late 50s)

## Discussion

Our study found a wide range of factors affecting the implementation and sustainability of interventions addressing oral health and related health behaviours among people experiencing SMD. By categorising the factors according to domains of the socioecological model,^20^ we attempted to provide a theoretical context to the study findings. The study found the need to reorient healthcare services for people experiencing SMD so that they acknowledge the inherent challenges of the people experiencing SMD better. Dedicated funding over a longer duration of time is essential to ensure specialist and sustainable health services are available for people experiencing SMD. Additionally, placing more trained staff, including support workers, can help provide a more targeted intervention for people experiencing SMD. Our findings emphasised the role of interpersonal factors (relationships between service providers and service users) as crucial in delivering and implementing health interventions. In addition to the presence of a positive relationship with the service providers, in terms of trust, mutual respect and non-judgemental communication, our study also found the need for the availability of training for healthcare providers. A systematic review of the global literature was conducted as part of our project to identify evidence on the implementation and sustainability of health interventions to improve oral health and related health behaviours in people experiencing SMD.^21^ This review reflects the findings of the present paper and finds moderate evidence that trust, resources and motivation levels are required to improve the implementation and sustainability of health interventions. Similarly, our findings related to training for staff were also found in a previous study that reported that ‘challenging behaviour’, of service users, often as a result of underlying social and health issues of people with SMD, can be a barrier in delivering the intervention.^22^

The location and time at which the health interventions are delivered to people experiencing SMD can make a difference in their implementation and sustainability. For instance, people experiencing SMD can find it challenging to access services very early in the mornings or later in the evenings, due to the timing of their jobs, probation appointments or even their preferences. Expecting people experiencing SMD to adhere to appointment times without understanding the inherent challenges faced by this group could set the interventions up for failure. A scoping review reiterates our findings and reports that rigid appointments were criticised in other studies and suggested that longer duration of appointments to address complex needs and flexible timings would make it easier for support workers and people experiencing homelessness to arrive at the health centres.^23^

At a more macrolevel, the intersection between health and housing has been widely and globally recognised as affecting people experiencing SMD.^24 25^ The interventions encompassing changes at the environmental, organisational and interpersonal levels will only be successful if they are implemented in cohesion with improving the social determinants of health. Housing-first interventions, especially those integrating housing with mental health interventions, have shown promise in helping people exit homelessness.^26^ Furthermore, issues related to oral health, other health conditions and health behaviours among people experiencing SMD were often dealt with and viewed separately by services and health professionals. However, these conditions often coexist and are interrelated. Unhealthy behaviours, such as smoking, alcohol and substance use, are associated with poor oral health^27 28^ and mental health conditions.^29^ There is also a link between mental health conditions and poor oral health status,^30^ which collectively are also linked to other chronic diseases.^31^ In such an overlapping plethora of disadvantages, there is a need to create an integrated pathway of care that addresses the health conditions of people experiencing SMD in a cohesive manner. The transient nature of people experiencing SMD, in terms of lack of fixed address, being in and out of prison and the complexities brought upon by mental health conditions and substance use—all need to be considered to make the interventions more implementable and sustainable. Recent National Institute for Health and Care Excellence guidelines recognise the need for targeted approaches and integrated multidisciplinary health and social care services for people experiencing homelessness, underpinned by co-design and co-delivery of services for sustained engagement with service users.^32^ Having trained staff could help with better patient preparedness, given that people with SMD can often present with multiple health conditions at the same time and may not necessarily have the same level of communication skills as some of the other patients. It is also important to give due importance to health promotion and preventative services in order to reduce the burden of unhealthy behaviours in people experiencing SMD. This has also been reflected in other UK and global literature, with studies finding that improved awareness about maintaining health and knowledge about the availability of interventions encouraged behaviour change through the active involvement of people experiencing SMD.^33 34^

### Strengths and limitations

Our study brings to the forefront the need to inclusively tackle multiple disadvantages faced by SMD groups to improve their healthcare. By interviewing a large number of practitioner stakeholders across England, we attempted to cover the extensive experiences of people planning and delivering the interventions. Our study also captured insights from people experiencing SMD, who are often underrepresented in research. We involved their participation from the early stages of the project in setting up the study documents and recruitment strategies, as well as by including them as study participants. The themes and results generated from our study hence explored the perspectives of service providers and service users, ensuring that the recommendations represent the experiences of both. We also had multiple interviewers speak with both groups of participants. This ensured there was flexibility in the way the questions were asked and we could eliminate the chances of bias from any single interviewer. A limitation of our study was that we recruited fewer people with experience of SMD than service providers, which could have risked the themes being more defined by the experiences of service providers. We have, however, attempted to assess the themes from each participant group's perspective individually in order to draw comparisons and ensure representation from service providers as well as people with experience in SMD. We also acknowledge that there is limited representation in the sample of people experiencing SMD from ethnic minorities or non-English-speaking backgrounds and from regions in England other than North East England. Our sample included fewer females experiencing homelessness as compared with males. Previous evidence has shown that women often experience more hidden forms of homelessness and are therefore not as present in the same organisations or services that were used to recruit men.^35^ Females, ethnic minorities and non-English-speaking people experiencing homelessness might face unique challenges (such as less knowledge of services, challenges in accessing health interventions and barriers due to language), which merits further research. We found that people who were in the midst of experiencing challenging situations related to SMD provided less depth of data. Nonetheless, those further in the recovery journey were able to reflect on and provide useful insights. While some themes in our results, such as integrated and cohesive services, were more defined by perspectives about support for mental health and substance use, the same can also be extrapolated in relation to oral health and smoking cessation services. The benefits of integrating smoking cessation services with oral health have long been discussed.^36 37^ In addition, our study found very little information related to the implementation of diet-related interventions, which reflects the general paucity of interventions addressing poor diet in this population.

### Implications and conclusions

We found that dedicated funding, integrated and inclusive services, trained healthcare providers, health promotion services and buy-in from service providers are important for the implementation and sustainability of interventions for oral health and substance use in people experiencing SMD.

Health interventions for people experiencing SMD need to be tailored or distinct from those that are aimed at the general population. A ‘one-size-fits-all’ approach to health and social care for people experiencing the combined effects of homelessness, substance use and repeat offending creates challenges as it does not account for the various socioeconomic difficulties of the SMD population. Treating homelessness as a public health issue, as well as a societal issue, is needed to reduce some of the disparity experienced by people experiencing SMD. Further research is needed to strengthen the evidence base for improving health outcomes for people experiencing SMD. This could focus on intervention development, co-producing interventions with people with experience of SMD and the evaluation of the implementation of interventions looking to improve oral health but also other aspects of health behaviours, especially interventions for improving diet.

## supplementary material

10.1136/bmjph-2023-000626 online supplemental file 1

10.1136/bmjph-2023-000626 online supplemental file 2

## Data Availability

No data are available.
