# Supplementary material for: Factors affecting implementation of interventions for oral health, substance use, smoking and diet for people with severe and multiple disadvantage: a community-based qualitative study in England
Source: BMJ Public Health. 2024 May 2;2(1):e000626. doi: 10.1136/bmjph-2023-000626 (PMC11812827; doi:10.1136/bmjph-2023-000626)
Supplement: online supplemental file 1 [file bmjph-2-1-s001.pdf]

Appendix 1

Factors affecting implementation of interventions for oral health, substance use, smoking and diet for people with severe and multiple disadvantage: a community-based qualitative study in England

Topic Guide: Interviews with people with experience of SMD

What works well in support services (general opening warm-up question)

To start with let's talk about what you feel works well with support services.

1. *Can you give me an example of when you have been supported by a service and it has really worked well for you? [This could be with your health or support with another issue like housing or probation for example].*
    1.1. *What was it that worked well for you?*
    1.2. *Why does this stand out against other experiences?*

If participant uses an example that includes oral health, smoking, substance misuse or diet then move to that section first.

Teeth and mouth problems

Let's move on to ***teeth and mouth issues****…*

2. *Have you ever had any problems with your* ***teeth and mouth****? This could be to do with teeth, an injury or a concern about something relating to your mouth – especially at a time when you were facing difficulties with homelessness or offending or drugs/alcohol.*

If yes, go to question 3.
If no, then probe once more: *have you needed to see a dentist or have any teeth issues like pain?*
If no, then go to questions on drugs/ alcohol.

[What worked well?]
3. *When you have had issues with your teeth and mouth, can you think of any experiences or treatment that worked well for you?*

Probe: What was good about it? Did you get the help you needed? When did you receive the support? Who provided the support? Where was it given?

If participant can't think of any positive experience –
3.1. *Have you ever had any help* ***for teeth or mouth issues*** *for you? This could be from dentists.*
If yes – *Did anything work well then? did you get the help you needed?*
3.2. If never had any help – *can you tell me a little about why that was?*
What would have made it easier to get support? Or, was there anything that made it hard for you to get support? So, what would have helped you?

[What did not work well?]
4. *Can you tell me a bit about a time when support or treatment didn't work so well or when you could not get the treatment you needed when you had issues with your teeth and mouth?*

Probe: *What did not work for you? What happened? What was difficult about that?*

[What needs to change]

5. *So, following on from these difficulties, what do you think needs to change or – if we wanted to see more people using services to help with* ***teeth and mouth issues****, what do you think could be done differently? What needs to change?*
   5.1. *Are there specific things that would make a real difference – is it about who provides the help; and who might that be? [Probe: Sometimes people with similar experiences as you can give help.]*
   5.2. Is it also about where the help/support is provided? [probe: looking back to times when you needed help, where would you have liked the help/support to be? – any particular locations? For example, in hostels, prison?

Drugs and alcohol

6. Can we now talk about ***drugs and alcohol issues****…Do you mind if I ask if you have ever had any* ***drug or alcohol*** *issues?* N.B.: could say 'drinking' instead of alcohol if participant uses 'drinking'.

If no – *go to questions on smoking.*

[What worked well?]
7. *Can you think of any services or support that worked well for you when you had issues with drugs and alcohol? Or maybe one particular service was better than others?*
Probe: What was good about it? Did you get the help you needed? When did you receive the support? Who provided the support? Where was it given?

[if participant can't think of any positive experience, go to question 5.1
7.1. Have you ever had any help ***for drug and alcohol issues****? This could be CBT, rehab or prescribing.*
7.1.1. If yes – *Did anything work well then? did you get the help you needed? When did you receive the support? Who provided the support? Where was it given?*
7.1.2. If no (i.e. never had any help) – *can you tell me a little about why that was? What would have made it easier to get support? Or, was there anything that made it hard for you to get support? So, what would have helped you?*

[What did not work well?]
8. *Can you tell me a bit about a time when support for drugs or alcohol didn't work so well or when you could not get the treatment you needed?*
Probe: *What did not work for you? What happened? What was difficult about that?*

[What needs to change]
9. *So, following on from these difficulties, what do you think needs to change or – if we wanted to see more people getting help with* ***drug and alcohol issues****, what do you think needs to change? What needs to be done differently?*
   9.1. *Are there specific things that would make a real difference – is it about who provides the support for drugs and alcohol; and who might that be? [Probe: Sometimes people with similar experiences as you can give help.]*
   9.2. Is it also about where support for drugs/alcohol can be provided? [probe: looking back to times when you needed help, where would you have liked the help/support to be? – any particular locations? For example, in hostels, prison?

Smoking

*Can we talk about some of these questions for **smoking**.*

10. *Do you mind if I ask if you have ever **smoked cigarettes or tobacco**?* If no, go to questions on diet.

11. [What worked well?]
    - *11.1. Have you ever tried to get help/support for **stopping smoking**?*
    - *11.2.* If yes – *Where did you go? Who did you get help from?*
    - *11.3. Have you had any support that worked well for you? What was good about it? Where did you go? Who gave you that support? Did you manage to get the right help/support?*

    - *11.4.* If no to 11.1 (i.e. never got help/support) – *can you tell me a little about why that was? What would have made it easier to get help? Or, was there anything that made it hard for you to get support? So, what would have helped you? Where would you go for help or who would you ask?*

12. [What didn't work well?]

Just thinking now about what hasn't work so well…

- *12.1. Were there times when you tried and could not get the help/support you needed to **stop smoking**?*
- *12.2. Has there been a time when the support didn't work for you?*

Probe: *What did not work for you? What happened? What was difficult about that?*

[What needs to change]

13. So, if with these difficulties, what needs to change?

- *13.1. If we wanted to see more people getting help to **stop smoking**, what do you think needs to change? What can be done differently?*
- *13.2. Are there specific things that would make a real difference – is it about who provides the help/support; and who might that be?* [Probe: Sometimes people with similar experiences as you can give help.]
- *13.3. Is it also about where the help/support is provided?* [probe: looking back to times when you needed help, where would you have liked the help/support to be? – any particular locations? For example, in hostels, prison?]

Sugary food and fizzy drinks

***Finally, can we talk about diet**.*

*14. Has there been a time when your diet has not been healthy, especially with **eating a lot of sugary foods and fizzy drinks**? This could've been sugars in tea/coffee, biscuits, or caffeinated drinks. This is again at a time when you were facing difficulties with housing (or offending).*

If no, go to question 15.

- *14.1.*If yes, *why was that? What led to that?*
- *14.2.What might have helped you not get into that habit?*
- *14.3.What could have helped stop this getting worse?*

15. Have you had any support services (maybe where you were staying), which was about healthy eating? Probe: *for example, classes on cooking or healthy eating?*

- *15.1.If yes – did you find this useful? What was good about it?*
- *15.2.What was not good about this?*

16. How could support services help people have less sugary food and drinks? What can help improve diet?

[General questions]

17. *We've talked about lots of different issues and difficulties (e.g. housing difficulties, teeth issues, drugs/ alcohol, high sugar diet, smoking) – looking back, did these start together or did one lead to another?* [Probe: So for example, did the job lead to housing, then drug use, homelessness.]

18. *Looking back to when you first identified that you needed help or treatment, do you know when it started? Say for teeth issues? When did it start? What could have stopped it from starting or getting worse?*
What about drug/ alcohol or smoking – when did that start? What started off that problem? What could have stopped it from starting?

19. *When we started the interview, you gave an example of when support/services have worked well to help you. Are there any ways that services for* ***teeth and mouth, substance misuse, diet or smoking could learn from that experience?*** Probes: *Could services work together or link up better? Are there better ways reaching out to people? Could support be changed to work better for you (flexibility)? If so, in what ways does it need to change?*
N.B.: further probes **only if needed: *Could we use peers (people with similar experiences)? Do you think staff training would help)?*

20. *We have spoken a lot about when you have or have not been able to get that support. Are there any specific times when getting that support was really critical or would have really helped? This could be a time you got the right support or when you wished you got that help – say for smoking, drug/alcohol? Probes: critical time points, in prison, in hostels, while getting support for something else, etc.*

21. *What would help people to take better care of their health? For example, brushing your teeth and looking after your mouth? And, what about better diet, less sugar? What might have helped?*

22. *What do you think are the most important changes that need to happen, so that people can get better support for these health issues? Any top 3 changes? Again, for people facing homelessness, substance use, and offending.*

23. *Is there anything you would like to add that you have not had the chance to say?*
