# Supplementary material for: Factors affecting implementation of interventions for oral health, substance use, smoking and diet for people with severe and multiple disadvantage: a community-based qualitative study in England
Source: BMJ Public Health. 2024 May 2;2(1):e000626. doi: 10.1136/bmjph-2023-000626 (PMC11812827; doi:10.1136/bmjph-2023-000626)
Supplement: online supplemental file 2 [file bmjph-2-1-s002.pdf]

Appendix 2

Factors affecting implementation of interventions for oral health, substance use, smoking and diet for people with severe and multiple disadvantage: a community-based qualitative study in England

Topic Guide: Interviews with service providers (e.g. frontline workers, practitioners, managers)

What works well in support services

To start with let's talk about what you feel works well with support for people facing multiple disadvantage.

1. *Can you give me an example from your own work or of another service that you think works well for people facing multiple disadvantage?*
    - *1.2. What is it that works?*
    - *1.3. Why does this stand out against other examples?*

If participant uses an example that includes oral health, smoking, substance misuse or diet then focus on this topic area first.

2. *[Oral Health] What do you do when you have someone you are supporting who has issues with their teeth and mouth? This could be dental issues, an injury or a concern about something relating to their mouth.* [N.B.: this is aimed more at frontline staff who are not dentists. For dentists, this could be worded slightly differently: *how do you support people facing multiple disadvantage who have dental issues?*]

3. *Can you tell me a little bit about any example of strong or inclusive services around **oral health / substance use/ smoking / diet*** [depending on participant's remit] *for people facing multiple disadvantage?*
   Why are these services effective? What models do they use? What makes them acceptable? Probe: what is it about these services (i.e. location, types of service, who delivers, etc)? examples of coproduction or service design that really stand out?
   **N.B.: If talking about drug/ alcohol here, then also probe Q 4.1 here.
   If talking about smoking here, then also probe Q 4.2 here.

4. *We've discussed about **dental health** [*or it could be drugs/alcohol] *are you aware of similar examples for drugs & alcohol/dental health? ? And what could be improved?* [N.B.: if discussion so far has been generic probe on any specific input related to dental health, drugs/alcohol, smoking/diet]

    4.1 [Drug/Alcohol] *Do you feel that you know how to support people effectively with drug and alcohol issues? Have you had any training? What is the provision of support for drug and alcohol like in your area?*

    4.2 [Smoking] *Do you know what smoking cessation services are available in your local area? Have you ever signposted people to smoking cessation services? What do you think would encourage the people you support to stop smoking or to get support? Where would you tell people to go to get that support?*

4.3 [Healthy Eating?] *Are you aware of any services locally to you which encourage healthy eating? Probe: for example, classes on cooking or healthy eating? Have you ever delivered support to try and improve diet with the people you support? This could be cooking lessons or help to purchase healthy food. Did it work? Why is that/What enables people to have a healthy diet?*

General Questions

5. *What could be improved about provisions locally/nationally to support people facing multiple disadvantage with their health?*

6. *What would make services more responsive and usable for people facing multiple disadvantage? How can services adapt to work better with the people you support?*

7. *Are there specific things that could make a real difference, say for **oral health* [depending on participant's area/remit] – *is it about* ***who*** *provides the help; and who might that be? Is it also about* ***where*** *the help/support is provided? [probe: any particular locations? For example, in hostels, prison?]; Are there any specific times or circumstances* ***when*** *it would be most beneficial? Probes: critical time points, in prison, in hostels, while getting support for something else, etc.*
   You mentioned what would make a real difference for [oral health], are there things that might make a real difference for the other areas we are exploring [drug & alcohol use, smoking, diet]?'.

8. *Are there any ways that services for* ***teeth and mouth, substance misuse, diet or smoking could be better integrated or linked up?*** Probes: Could there be better coordination/collaboration?

9. *What do you think are the most important changes that need to happen, so that people can get better support for these health issues?* [N.B.: trying to get at range of factors for improving access, or prevention, including wider determinants e.g. housing, etc.]

10. *Is there anything you would like to add that you have not had the chance to say?*

Closing: *thank you so much for your time today. If you'd like to hear more about the study, please let me know. We are planning a workshop to share findings with stakeholders, if you'd be interested in joining, we can send you details.*
